# Somatosensory‐Thalamic Functional Dysconnectivity Associated With Poststroke Motor Function Rehabilitation: A Resting‐State fMRI Study

**DOI:** 10.1002/brb3.70321

**Published:** 2025-02-11

**Authors:** Haojie Zhang, Jun Zhao, Lingzhong Fan, Chaohong Gao, Fang Li, Jingya Liu, Chen Bai, Xingzhu Li, Bingjie Li, Tong Zhang

**Affiliations:** ^1^ School of Rehabilitation Medicine Capital Medical University Beijing China; ^2^ Center of Neurological Rehabilitation China Rehabilitation Research Center Beijing China; ^3^ China Rehabilitation Science Institute Beijing China; ^4^ Department of Neurology China Rehabilitation Research Center Beijing China; ^5^ Brainnetome Center and National Laboratory of Pattern Recognition, Institute of Automation Chinese Academy of Sciences Beijing China; ^6^ Sino‐Danish College University of Chinese Academy of Sciences Beijing China; ^7^ Sino‐Danish Center Beijing China; ^8^ Department of Occupational Therapy China Rehabilitation Research Center Beijing China

**Keywords:** functional connectivity, neuroplasticity, rehabilitation, stroke, thalamus

## Abstract

**Background:**

The thalamus plays a pivotal role in functional brain networks, yet its contribution to motor function recovery following stroke remains elusive. We aim to explore changes in thalamocortical functional connectivity poststroke and its correlation with motor function.

**Methods:**

Thirty‐nine subacute ischemic stroke patients and 32 healthy individuals underwent resting‐state functional magnetic resonance imaging (MRI). The Fugl–Meyer Assessment (FMA) was employed to evaluate upper and lower extremity motor function before and 1 year after stroke rehabilitation. The ipsilesional thalamus and contralesional thalamus were parceled into functional regions of interest (ROIs) based on connectivity with six cortical ROIs: prefrontal, motor, temporal, posterior parietal, somatosensory, and occipital cortex. Functional connectivity between each cortical ROI and its corresponding thalamic ROI was calculated and compared between groups. Differences identified in the ROI‐to‐ROI analysis were further investigated through seed‐to‐voxel whole‐brain connectivity analyses to pinpoint thalamic dysconnectivity. Correlations with upper and lower extremity motor function were also analyzed.

**Results:**

Significant changes in thalamocortical functional connectivity were observed after stroke in ROI‐to‐ROI analysis, with bilateral somatosensory‐thalamic connectivity decreased and ipsilesional temporal‐thalamic and bilateral occipital‐thalamic connectivity increased. Seed‐to‐voxel analysis localized ipsilesional thalamic hypoconnectivity to the ipsilesional rolandic operculum and ipsilesional precentral gyrus. Ipsilesional somatosensory‐thalamic connectivity was positively correlated with baseline upper extremity FMA scores and negatively correlated with upper extremity motor function change rate at 1‐year postdischarge.

**Conclusions:**

This study provides new insights into the role of the thalamus in motor function recovery after stroke, offering preliminary evidence for its potential as a therapeutic target in poststroke rehabilitation.

## Graphical abstract

Combining ROI‐to‐ROI and seed‐to‐voxel approaches, we conducted an in‐depth analysis of thalamocortical functional dysconnectivity. Our findings provide new insights into the role of the thalamus in motor recovery following stroke, offering preliminary evidence that suggests the thalamus may serve as a potential therapeutic target for poststroke motor rehabilitation.

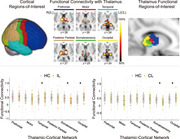

## 1 Introduction

Stroke is a leading cause of adult disability worldwide, generating a substantial demand for rehabilitation (Tu et al. 2023; Feigin and Owolabi 2023; Tu and Wei 2024). The mechanisms of brain plasticity serve as the theoretical basis for rehabilitation, and a comprehensive understanding of poststroke brain plasticity can effectively guide rehabilitation strategies aimed at reducing disease burden (Grefkes and Fink 2020; Bernhardt et al. 2017). Research on motor rehabilitation poststroke has mainly focused on structural and functional changes in motor‐related brain regions, such as the corticospinal tract (Lim et al. 2020; Soulard et al. 2020) and motor areas (Sanders et al. 2022; Hensel et al. 2023). However, stroke is complex and heterogeneous, making brain plasticity mechanisms equally intricate (Langhorne et al. 2011). Prediction models using motor system‐related neuroimaging biomarkers and clinical characteristics offer valuable insights but only partially explain motor recovery outcomes (Plantin et al. 2021; Kim and Winstein 2017). Thus, structural and functional changes in nonmotor areas may also significantly influence motor recovery (Choudhury et al. 2019; Baker et al. 2015). Studies have shown that interhemispheric sensorimotor network connectivity is associated with upper extremity motor function (Carter et al. 2010; Liu et al. 2022), and greater activation in nonmotor areas is generally linked to poorer functional recovery (Calautti and Baron 2003). These findings highlight the importance of considering broader brain network changes beyond motor‐specific regions in poststroke recovery.

The thalamus, a sensory relay and crucial brain network hub (Hwang et al. 2021; Ward 2013), holds potential significance in poststroke motor recovery. It connects the periphery to the cerebral cortex and different cortical areas (Usrey and Sherman 2019), integrating information flows that impact motor network plasticity (Kawaguchi 2017; Aswendt et al. 2021; Balbinot and Schuch 2018). Proprioceptive neuromuscular facilitation principles suggest that increased proprioceptive input enhances motor cortex output, implying the thalamus's critical role in motor rehabilitation (Shimura and Kasai 2002). Studies show that functional connectivity between the ipsilesional primary motor cortex and contralesional thalamus correlates positively with motor recovery (Park et al. 2011). However, previous analyses treated the thalamus as a whole, not exploring specific subregion changes.

This study aims to investigate changes in thalamocortical functional connectivity after stroke and their correlation with motor function, focusing on patients with subacute subcortical infarcts sparing the thalamus. Using functional connectivity analysis, we parcellated both the ipsilesional thalamus (IL_‐Tha_) and contralesional thalamus (CL_‐Tha_) into distinct functional subregions. This approach allows for a more nuanced examination of thalamic subregion connectivity patterns, potentially providing deeper insights into the brain plasticity underlying motor recovery. We hypothesize that thalamocortical functional connectivity differs between patients (IL_‐Tha_, CL_‐Tha_) and healthy controls (HCs), correlating with motor rehabilitation outcomes.

## 2 Methods

### 2.1 Participants and Study Design

Thirty‐nine subacute subcortical ischemic stroke patients and 32 age‐matched HCs were recruited in this study. Inclusion and exclusion criteria were detailed in the Supporting Information.

A prospective longitudinal observational study was conducted. Enrolled patients underwent one resting‐state functional magnetic resonance imaging (rs‐fMRI) scan and clinical assessments before rehabilitation, followed by 1–2 months of standard rehabilitation treatment based on principles consistent with the American Heart Association/American Stroke Association (AHA/ASA) guidelines for adult stroke rehabilitation (Winstein et al. 2016) at China Rehabilitation Research Center, including physical and occupational therapies (≥3 h/day, 5 days/week). After discharge, rehabilitation continued in the community or at home, and we developed an individualized rehabilitation program for each patient. Motor function improvement at 1 year after discharge was evaluated at follow‐up. The HCs completed one rs‐fMRI scan.

The study was approved by the Ethics Committee of the China Rehabilitation Research Center (2018‐008‐1), and each participant signed an informed consent form after receiving detailed information about the study.

### 2.2 Clinical Assessment and Quality Control

Considering potential differences in neuroplasticity mechanisms for upper and lower extremity motor function recovery (Lee et al. 2021; Peters et al. 2021), we used the Fugl–Meyer Assessment (FMA) to assess patients' upper and lower extremities independently. The upper extremity assessment included 33 items, while the lower extremity assessment included 17 items, with scores ranging from 0 to 2 for each item. The maximum scores were 66 for the upper extremity and 34 for the lower extremity, with higher scores indicating better motor function. Stroke severity was assessed using the National Institutes of Health Stroke Scale (NIHSS), consisting of 11 items, with a total score range of 0–42; higher scores indicated more severe neurological damage.

Rehabilitation outcomes were evaluated using the change rate of motor function (CR‐MF), calculated as follows:

$$\begin{array}{ccc} \mathrm{CR}-\mathrm{MF} & = & \left(\mathrm{FM}A_{-1\mathrm{year}}-\mathrm{FM}A_{-\mathrm{baseline}}\right)/\\ & & \left(\mathrm{FM}A_{-1\mathrm{year}}+\mathrm{FM}A_{-\mathrm{baseline}}\right) \end{array}$$

This method aims to control for the “ceiling effect” of the FMA scale and balances the effect of the patient's baseline values (Zhang et al. 2024).

To ensure research quality and minimize confounding factors, the imaging data were independently processed by an imaging specialist who was blinded to the clinical information, while clinical rehabilitation assessment physicians remained blind to the imaging data results.

### 2.3 Analytical Strategy of Imaging

The imaging analysis included two main steps. First, six nonoverlapping cortical regions of interest (ROIs) were defined, and a winner‐take‐all approach partitioned IL_‐Tha_ and CL_‐Tha_ into six corresponding functional subregions. Functional connectivity between each cortical ROI and its thalamic counterpart was compared across groups. Second, using thalamic ROIs with significant connectivity differences identified in the first analytical step as seeds, seed‐to‐voxel whole‐brain connectivity comparisons examined the connectivity of specific thalamic subregions with the rest of the brain. This strategy highlights the specific regional characteristics of the cerebral cortex and thalamus (Woodward and Heckers 2016).

### 2.4 MRI Data Acquisition and Preprocessing

All images were acquired on a Philips Ingenia 3.0T TX magnetic resonance scanner (Royal Philips Electronics, Eindhoven, the Netherlands) at the China Rehabilitation Research Center. Scan parameters were detailed in the Supporting Information.

Before preprocessing, image quality and basic parameter information for each subject were manually checked by the imaging specialist. To facilitate group comparisons, the images from patients with left hemisphere lesions (*n* = 17) were flipped along the midsagittal plane to make the lesioned side correspond to the right hemisphere in all subjects. Accordingly, considering potential systematic hemispheric asymmetry, the data from 32 HCs were also flipped to create a 64‐subject HC dataset (Hensel et al. 2023; Liu et al. 2020).

The imaging data were preprocessed using MATLAB version 2013b (The Mathworks Inc., MA, USA) with loaded Statistical Parametric Mapping (SPM version 12; http://www.fil.ion.ucl.ac.uk) and BRAinNetome Toolkit (BRANT version 3.36; http://www.brainnetome.org). The preprocessing procedure included discarding the first five functional volumes, correction for slice‐timing, and head motion, excluding data with head motion > 3 mm in any direction. T1_3D high‐resolution structural images were used to normalize the functional images to Montreal Neurological Institute (MNI) space through linear and nonlinear transformation. Denoising included linear detrending, filtering (0.01–0.08 Hz), and regression of covariates including motion parameters, white matter, and cerebral spinal fluid signals. Notably, whole‐brain global signal regression was not performed here, as evidence suggests it may compromise data validity (Saad et al. 2012). Consistent with previous related studies, no spatial smoothing was applied at this stage (Woodward, Karbasforoushan, and Heckers 2012).

### 2.5 Cortical ROI Definition

**FIGURE 1:**
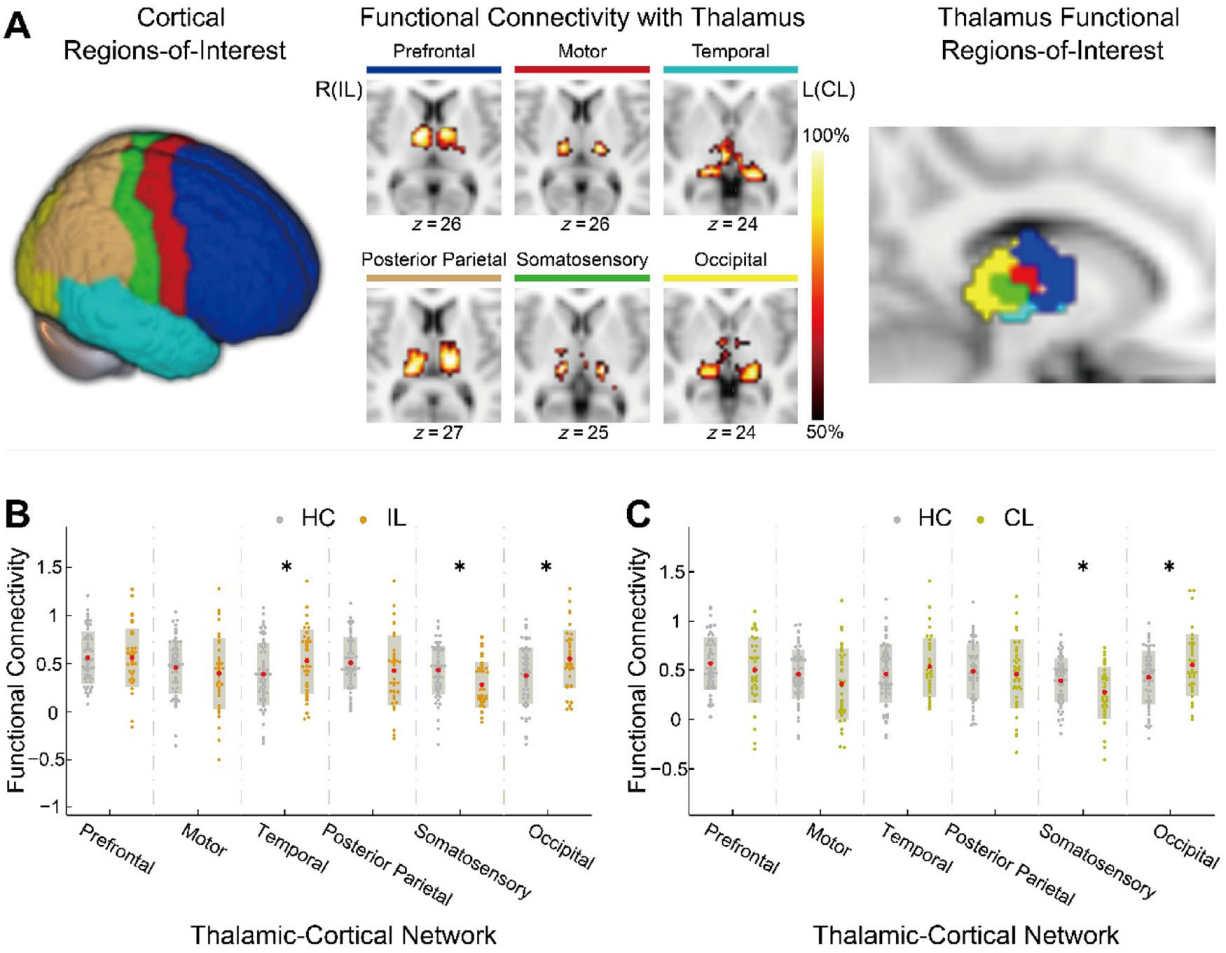
Subregion parcellation of thalamus and thalamocortical functional connectivity differences between groups. (A) Based on a priori defined regions of interest (ROIs) in the cerebral cortex and the winner‐takes‐all approach for functional connectivity, ipsilesional thalamus (IL_‐Tha_) and contralesional thalamus (CL_‐Tha_) were parceled into six specific, largely nonoverlapping functional subregions, respectively. The color bar represents the 50%–100% maximum correlation coefficient values (Zhang et al. 2010). (B) and (C), respectively, depict intergroup differences in cortical ROI–thalamic ROI connectivity for the IL_‐Tha_, CL_‐Tha_, and healthy controls (HCs). Gray dots represent the HC, orange dots represent the IL_‐Tha_, and light green represents the CL_‐Tha_. “*” indicates significant differences (*p* < 0.05). We adopted a presentation format as Woodward and Heckers (2016) to facilitate comparison of the current results with previous findings. L, left; R, right.

Referring to previous studies on thalamus segmentation (Woodward and Heckers 2016; Hale et al. 2015), we utilized the refined Brainnetome Atlas (http://atlas.brainnetome.org/index.html; Fan et al. 2016) to divide the cerebral cortex into six ROIs and extract bilateral thalamus masks. (1) Prefrontal: including superior frontal gyrus, middle frontal gyrus, inferior frontal gyrus, and orbital gyrus; (2) Motor: including precentral gyrus and paracentral lobule; (3) Temporal: including superior temporal gyrus, middle temporal gyrus, inferior temporal gyrus, fusiform gyrus, parahippocampal gyrus, and posterior superior temporal sulcus; (4) Posterior parietal: consisting of superior parietal lobule, inferior parietal lobule, and precuneus; (5) Somatosensory: consisting of postcentral gyrus; (6) Occipital: consisting of medioventral occipital cortex and lateral occipital cortex (Figure 1).

### 2.6 Thalamus Parcellation and Functional Connectivity Analysis

Utilizing MATLAB version 2013b, we conducted functional connectivity analysis and thalamus parcellation. Within the dataset of 103 samples including stroke patients and HCs, the average blood oxygenation level‐dependent (BOLD) signal time series was extracted from the unsmoothed functional data for each cortical ROI. Pearson correlation analysis was then conducted between the BOLD signal time series of each voxel in the bilateral thalamus and that of the cortical ROIs (Woodward, Karbasforoushan, and Heckers 2012; Hale et al. 2016). Ipsilesional thalamus (IL_‐Tha_) and contralesional thalamus (CL_‐Tha_) were parceled into six corresponding functional ROIs using a winner‐take‐all approach. This approach classified each voxel in the thalamus based on its strongest connection to a cortical ROI. Each subject generated an individualized winner‐take‐all thalamus parcellation map, which was then averaged across subjects to produce a group‐level winner‐take‐all thalamus parcellation map (Figure 1).

Based on the group‐level winner‐take‐all map, the functional connectivity between each cortical ROI and its corresponding thalamic ROI was calculated for the IL_‐Tha_, CL_‐Tha_, and HC group, with the HC group including bilateral thalamocortical functional connectivity. Furthermore, for purposes of calculating statistical significance, Pearson correlation coefficients were converted to a normal distribution using Fisher's *r*‐to‐*z* transform (Zhang et al. 2008).

### 2.7 Statistical Analysis

The statistical analysis was conducted in three main parts. First, two‐sample independent *t*‐tests were employed to examine differences in cortical ROI–thalamic ROI functional connectivity between stroke patients (IL_‐Tha_, CL_‐Tha_) and HC, with a significance level of 0.05 (two‐tailed). The purpose of this analysis was to investigate variations in functional connectivity between cortical ROIs and specific thalamic subregions. Second, thalamic ROIs exhibiting significant differences in the ROI‐to‐ROI analysis were selected as seeds for a seed‐based, voxel‐wise whole‐brain functional connectivity analysis. The significance level was set at 0.05, and the family‐wise error (FWE) correction was applied for multiple comparisons at the cluster level. This step aimed to delve into dysconnectivity between specific cortical regions and the thalamus. Finally, Pearson or Spearman correlation was used to scrutinize the relationship between cortical ROI–thalamic ROI functional connectivity and motor function.

## 3 Results

### 3.1 Clinical Characteristics

Demographic features of 39 patients closely matched with those of 32 healthy individuals, showing no significant differences in age, gender, body mass index, smoking habits, hypertension, diabetes, and dyslipidemia. The majority of patients were male (74.4%), primarily experiencing mild‐to‐moderate strokes (NIHSS: 6.4 ± 3.6, range: 0–14; van der Kemp et al. 2019), and were enrolled during the subacute phase within 1 month after stroke onset (20.1 ± 6.1 days). Lesion locations showed no significant lateralization differences between the two hemispheres (*p* = 0.26), indicating no hemispheric dominance effects when comparing patients to HCs. Both upper and lower extremity motor functions demonstrated substantial improvement at 1‐year postdischarge (*p* < 0.001; Table 1).

**TABLE 1:** Clinical characteristics.

	Patients (*n* = 39)	Healthy controls (*n* = 32)	*t/χ^2^*	*p*
Age, mean (range)	57.7 (27–74)	52.5 (25–71)	1.96	0.054
Male, *n* (%)	29 (74.4)	24 (75.0)	0.004	0.951
BMI, mean (±SD)	26.1 (±3.2)	25.2 (±3.5)	1.088	0.281
Smoke, *n* (%)	17 (43.6)	9 (28.1)	1.811	0.178
Hypertension, *n* (%)	27 (69.2)	19 (59.4)	0.748	0.387
Diabetes, *n* (%)	13 (33.3)	6 (18.8)	1.907	0.167
Dyslipidemia, *n* (%)	22 (56.4)	14 (43.8)	1.127	0.288
MMSE, mean (±SD)	29.4 (±1.1)	29.6 (±0.7)	0.949	0.346
Lesion side			1.282	0.258
Left, *n* (%)	17 (43.6)			
Right, *n* (%)	22 (56.4)			
Days poststroke, mean (±SD)	20.1 (±6.6)			
NIHSS			13.578	< 0.001
T1	6.4 (±3.6)			
T2	1.1 (±1.9)			
FMA‐UE			14.228	< 0.001
T1	22.6 (±16.0)			
T2	52.7 (±16.8)			
FMA‐LE			9.828	< 0.001
T1	21.9 (±8.2)			
T2	31.9 (±3.2)			

Abbreviations: BMI, body mass index; FMA‐LE, Fugl–Meyer Assessment of the lower extremity; FMA‐UE, Fugl–Meyer Assessment of the upper extremity; MMSE, mini‐mental state examination; NIHSS, National Institutes of Health Stroke Scale; SD, standard deviation; T1, evaluation at baseline; T2, evaluation at 1‐year postdischarge.

### 3.2 Cortical ROI‐to‐Thalamic ROI Analysis

As shown in Figure 1A, the group‐level thalamocortical connectivity maps were virtually consistent with prior studies that have parceled the thalamus based on its structural (Behrens et al. 2003; Zhang et al. 2010) and functional connectivity (Woodward, Karbasforoushan, and Heckers 2012; Hale et al. 2015). Each cortical ROI was connected to distinct, largely nonoverlapping regions of the thalamus, with the functional subdivisions on the IL_‐Tha_ and CL_‐Tha_ being generally symmetrical. Specifically, the prefrontal cortex exhibited strong functional connectivity with the anterior and dorsal regions of the thalamus, primarily involving the anterior nucleus group and the anterior part of dorsomedial nucleus. The motor cortical subdivision was functionally connected to the ventral lateral portion of the thalamus, presumptively involving the ventral lateral and ventral anterior nucleus. The temporal lobe connected with the medial, inferior, and posterior portions of the thalamus, possibly corresponding to the medial geniculate body, medial part of the pulvinar, and medial nuclear group. The posterior parietal cortex mainly shows correlations with the dorsal and posterior areas of the thalamus, which may contain the lateral posterior nucleus and the pulvinar. The somatosensory cortical ROI strongly connected the thalamic ventral, lateral, and posterior regions, possibly comprising the ventral posterior nucleus. The occipital lobe showed strong interactions with the posterior and lateral portions of the thalamus, presumptively corresponding to the lateral geniculate body and pulvinar.

Using a two‐tailed two‐sample *t*‐test, cortical ROI–thalamic ROI functional connectivity was compared between the IL_‐Tha_ and HC, as well as between the CL_‐Tha_ and HC. Bilateral somatosensory‐thalamic connectivity was significantly decreased compared to controls (*p* < 0.05), while ipsilesional temporal‐thalamic and bilateral occipital‐thalamic connectivity were significantly enhanced (*p* < 0.05). However, there were no significant differences in prefrontal‐thalamic, motor‐thalamic, and posterior parietal‐thalamic connectivity between the patient group and controls. See Figure 1.

### 3.3 Thalamus ROI Seed‐Based Analysis

To better localize thalamic dysconnectivity with the rest of the brain, seed‐to‐voxel whole‐brain connectivity analysis was performed using five thalamic subregions that showed significant differences in the ROI‐to‐ROI analysis as seeds. These seeds included bilateral thalamic somatosensory and occipital subregions, along with the ipsilesional thalamic temporal subregion. Intergroup comparisons were made between the patients (IL_‐Tha_, CL_‐Tha_) and the HC group, with FWE correction applied for multiple comparisons at the cluster level with a significance level of 0.05.

Functional connectivity was reduced between the IL_‐Tha_ somatosensory seed and ipsilesional regions including the rolandic operculum, precentral gyrus, cingulate gyrus, and postcentral gyrus. Connectivity was also reduced between the CL_‐Tha_ somatosensory seed and the ipsilesional postcentral gyrus and insula but increased with the contralesional cingulate gyrus.

Enhanced connectivity was observed between the IL_‐Tha_ occipital seed and the contralesional middle temporal gyrus and fusiform gyrus, while the whole‐brain connectivity result of the CL_‐Tha_ occipital seed did not meet significance after applying multiple comparisons correction at the a priori defined level.

**FIGURE 2:**
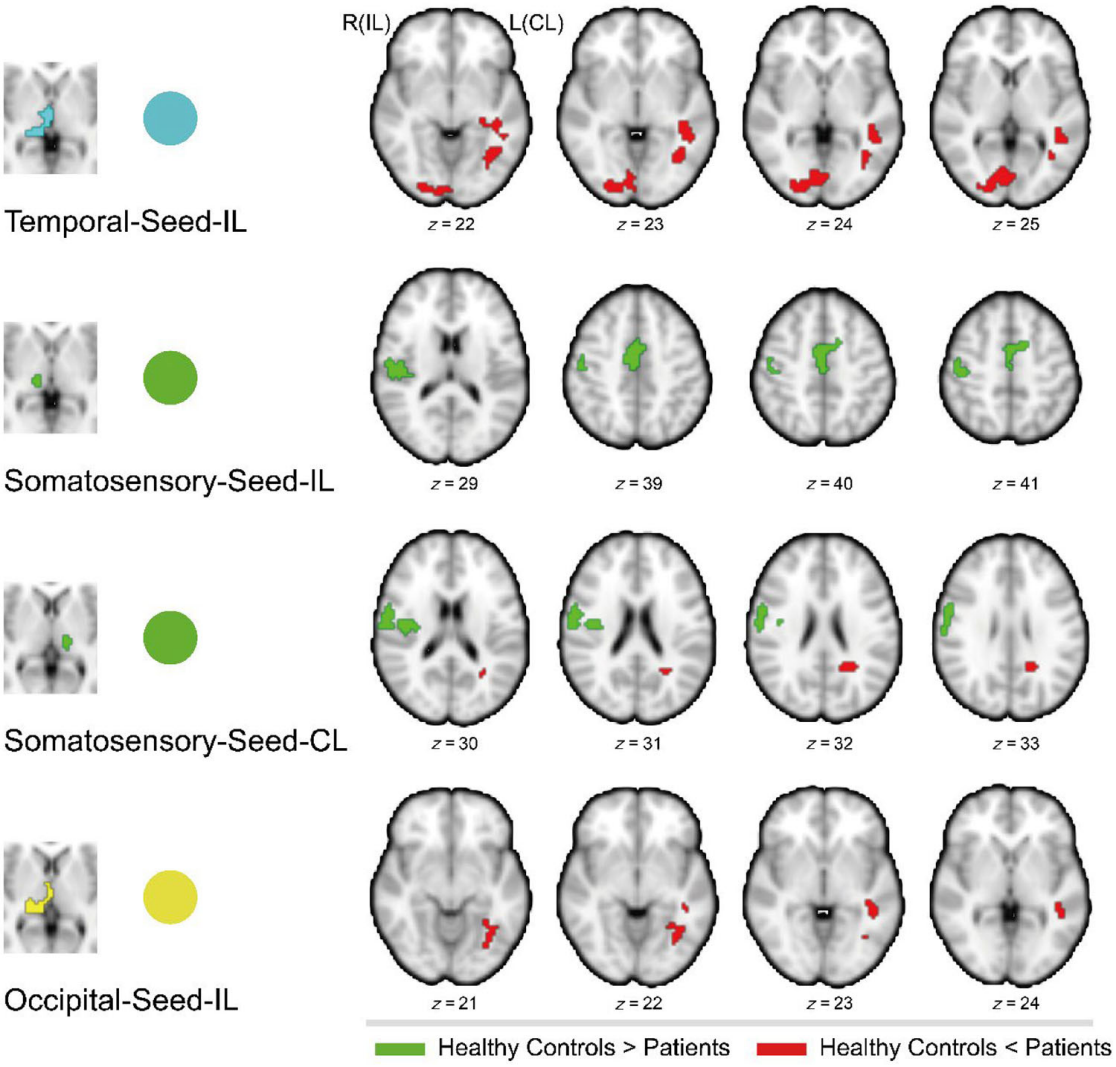
Intergroup differences in seed‐to‐voxel whole‐brain connectivity analysis. The four thalamic functional subregion seeds are shown on the left. On the right are cluster regions with significant differences between groups. Green clusters indicate reduced functional connectivity in stroke patients compared to healthy controls. Red clusters indicate enhanced functional connectivity in stroke patients compared to healthy controls. CL, contralesional; IL, ipsilesional.

For the IL_‐Tha_ temporal seed, its connectivity with the contralesional fusiform gyrus, middle temporal gyrus, and the ipsilesional middle occipital gyrus was enhanced. See Table 2 and Figure 2.

**TABLE 2:** Group differences in whole‐brain functional connectivity of thalamic seed regions.

				MNI				
Thalamus seeds	Contrast	Brain regions	Voxels	*x*	*y*	*z*	Peak *t*	*p*_(FWE‐cor)_
Temporal‐IL	IL > HC	L, fusiform gyrus	85	−36	−60	−6	4.85	< 0.001
				−36	−60	6	3.74	
				−42	−51	−12	3.3	
		L, middle temporal gyrus	92	−45	−42	0	4.64	< 0.001
				−30	−30	−6	3.91	
				−24	−30	−15	3.78	
		R, middle occipital gyrus	244	27	−90	−3	4.35	< 0.001
				18	−90	3	4.06	
				3	−81	3	4.04	
	HC > IL	*No significant clusters*						
Somatosensory‐IL	IL > HC	*No significant clusters*						
	HC > IL	R, rolandic operculum	82	48	−18	12	4.56	< 0.001
				57	−21	12	3.95	
				57	−6	9	3.67	
		R, precentral gyrus	195	0	−12	72	4.25	< 0.001
				6	−27	81	3.98	
				0	−39	78	3.87	
		R, cingulate gyrus	153	3	−9	45	4.16	< 0.001
				−12	3	54	3.99	
				0	0	51	3.9	
		R, postcentral gyrus	112	45	−21	57	4	< 0.001
				51	−15	48	3.93	
				42	−30	63	3.73	
Somatosensory‐CL	CL > HC	L, cingulate gyrus	37	−18	−51	24	3.99	0.002
				−27	−60	18	3.45	
	HC > CL	R, postcentral gyrus	519	60	−9	18	5.52	< 0.001
				57	0	24	5.27	
				54	−6	39	4.52	
		R, insula	65	42	−12	18	5.06	< 0.001
Occipital‐IL	IL > HC	L, middle temporal gyrus	36	−42	−36	−3	4.4	0.01
		L, fusiform gyrus	49	−33	−66	−9	4.21	0.02
				−30	−54	−6	3.52	
	HC > IL	*No significant clusters*						

Abbreviations: CL, contralesional, aligned with left hemisphere; FWE, family‐wise error; HC, healthy control; IL, ipsilesional, aligned with right hemisphere; L, left; MNI, Montreal Neurological Institute; R, right.

### 3.4 ROI–ROI Connectivity and Motor Function Correlation Analysis

**FIGURE 3:**
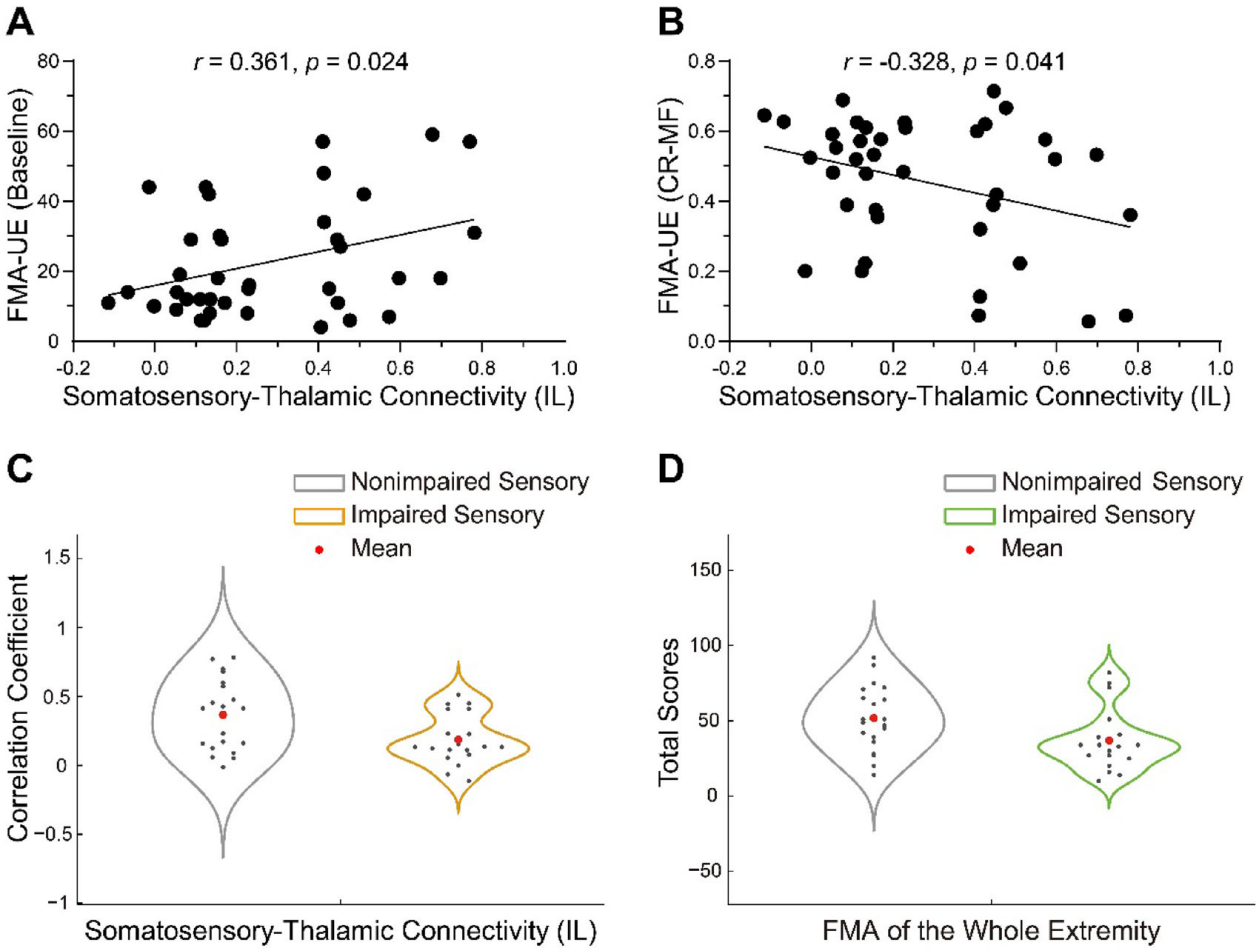
Correlation between ipsilesional somatosensory‐thalamic connectivity and upper extremity motor function. (A) depicts the correlation between ipsilesional somatosensory‐thalamic connectivity and baseline upper extremity FMA scores. (B) highlights the correlation between ipsilesional somatosensory‐thalamic connectivity and change rate of upper extremity motor function at 1‐year postdischarge. (C) and (D) represent the intergroup differences in ipsilesional somatosensory‐thalamic connectivity and FMA scores of the whole extremity between the impaired sensory group and the nonimpaired sensory group, respectively. CR‐MF, change rate of motor function; FMA‐UE, Fugl–Meyer Assessment of the upper extremity; IL, ipsilesional.

To examine the correlation between thalamocortical dysconnectivity and motor function, we conducted linear correlation analyses between the aforementioned five cortical ROI–thalamic ROI connectivity and both baseline motor function and the CR‐MF. Considering potential neuroplasticity differences between upper and lower extremities, separate correlation analyses were performed for the motor functions of the upper and lower extremities. The results revealed a positive correlation between ipsilesional somatosensory‐thalamic connectivity and baseline upper extremity FMA scores (*r* = 0.361, *p* = 0.024; Figure 3A) and a negative correlation with the CR‐MF for the upper extremity (*r* = −0.328, *p* = 0.041; Figure 3B).

To gain a deeper understanding of potential disparities in ipsilesional somatosensory‐thalamic connectivity between patients with sensory dysfunction and those with normal sensory function, we organized patients into groups based on the sensory assessment item in the NIHSS. This resulted in an impaired sensory group (*n* = 19) and a nonimpaired sensory group (*n* = 20), and an independent samples *t*‐test was conducted between the two groups. Furthermore, we analyzed potential motor function differences between the impaired and nonimpaired sensory groups. It is important to note that sensory impairment did not distinguish between upper and lower extremities; therefore, FMA scores encompassed a whole evaluation of both upper and lower extremities. The findings revealed that, in comparison to the nonimpaired sensory group, the impaired sensory group exhibited a significant reduction in ipsilesional somatosensory‐thalamic connectivity (*t* = 2.53, *p* = 0.016; Figure 3C), along with more pronounced impairment in motor function (*t* = 2.23, *p* = 0.032; Figure 3D).

## 4 Discussion

The thalamus plays a crucial role in the brain, yet its involvement in the stroke recovery process remains not fully understood. This study employed rs‐fMRI to investigate alterations in thalamocortical functional connectivity during the subacute period following a stroke and its correlation with motor function. We observed reduced bilateral somatosensory‐thalamic connectivity, while ipsilesional temporal‐thalamic and bilateral occipital‐thalamic connectivity increased. The ipsilesional somatosensory‐thalamic connectivity was associated with both the baseline and the rate of change in motor function for the upper extremity. Elucidating thalamocortical circuit changes poststroke may reveal crucial neuroplasticity mechanisms that can guide more targeted rehabilitation programs to improve motor outcomes.

Thalamus serves as a crucial hub in the brain functional network, divisible into distinct subregions, each with unique functions. This subcortical structure, composed of numerous nuclei, exhibits robust functional connections with various networks (Cole, Pathak, and Schneider 2010; Keser et al. 2021), encompassing motor, sensory, language, and cognitive systems. Typically, the medial thalamus is closely linked to the default mode network, while the visual and dorsal attention networks exhibit strong connectivity with the pulvinar. The cingulo‐opercular network demonstrates a robust connection with the ventral intermediate thalamus, and the frontoparietal network, somatomotor, and salience networks each connect functionally with corresponding thalamic subregions (Greene et al. 2020). In this study, utilizing the cortical ROI‐to‐thalamic ROI approach in a sample comprising patients with subcortical infarcts and healthy individuals, the thalamic functional parcellation map maintains high consistency with previous research findings, indicating that thalamocortical functional connectivity may be relatively stable. We speculate that functional connectivity between two regions derived from BOLD signal temporal correlation likely receives certain structural support, even though functionally connected areas do not necessarily have direct white matter connections. Furthermore, prior studies have shown that thalamic parcellation based on functional connectivity agrees well with the “gold standard” histological atlas (Hale et al. 2015), lending initial support for developing connectivity‐based rehabilitation protocols for stroke recovery.

The key findings of this study indicate dysconnectivity between the cortical somatosensory, occipital, and temporal regions with the thalamus following a stroke. Furthermore, the ipsilesional somatosensory‐thalamic dysconnectivity was associated with upper extremity motor function, suggesting the potential involvement of the somatosensory, visual, and auditory networks in brain plasticity during motor rehabilitation poststroke. Previous research found a correlation between functional connectivity of the interhemispheric somatosensory cortex and poststroke hand motor function. Paretic hand movement necessitates information exchange between the somatosensory cortex and precuneus to support visual processing, and complex motor tasks may involve the bilateral temporal lobes in auditory processing (Liu et al. 2022; Lacourse et al. 2005). Notably, somatosensory‐thalamic connectivity decreased while occipital‐thalamic and temporal‐thalamic connectivity increased, indicating their plasticity changes are not aligned. The reduction in somatosensory‐thalamic connectivity may be related to damage in ascending nerve fibers conveying sensory information between the thalamus and somatosensory areas, particularly in patients with severe motor impairment. Although explaining abnormal functional connectivity by anatomical damage is not entirely reasonable, there may be a structural basis for functional connectivity between regions as previously discussed (van Meer et al. 2010; Damoiseaux and Greicius 2009). Plasticity changes in somatosensory‐thalamus connectivity warrant further exploration. Regarding occipital, temporal, and thalamic connectivity, the thalamus plays a crucial integrative role in cognitive networks like the dorsal and ventral attention networks involving the occipital and temporal lobes. Cognitive networks may participate in motor execution and have predictive value for poststroke motor recovery (Cheng et al. 2021). However, this study did not observe significant correlations between occipital‐thalamic and temporal‐thalamic connectivity with motor function. In conclusion, the thalamocortical circuit emerges as a vital pathway for integrating sensory and motor signals. Exploring plastic changes in cortical‐thalamic connectivity may contribute to refining predictive models for poststroke motor function recovery (Carlson et al. 2020).

Combining ROI‐to‐ROI and seed‐to‐voxel methods enables a more specific analysis of functional connectivity differences between the cerebral cortex and thalamus. Using the ROI‐to‐ROI analysis, we segmented the thalamus into six specific functional subregions, but large cortical ROIs may obscure functional connectivity differences that exist in specific brain regions (Woodward, Karbasforoushan, and Heckers 2012). Employing the seed‐to‐voxel method, we identified numerous functional connectivity differences between specific brain regions and the thalamus. A notable difference involved a weakened connection between the ipsilesional precentral gyrus and the ventroposterior lateral areas of the IL_‐Tha_, suggesting abnormalities in the connectivity between the motor network and the thalamus. In contrast to our findings, Park et al. (2011) observed enhanced functional connectivity between the ipsilesional primary motor cortex and the bilateral thalamus. This inconsistency may be attributed to variations in sample characteristics, such as stroke duration and severity, across studies. Additionally, differences in seed selection may also be an important potential factor. While Park et al. calculated connectivity between the mid‐lower part of the precentral gyrus and the entire thalamus, we observed connectivity abnormalities specifically between the ventroposterior lateral region of the IL_‐Tha_ and clusters peaked in the precentral gyrus. Selecting appropriate ROIs for functional connectivity analysis is complex and critical, requiring substantial knowledge accumulation, and results may need verification through different studies across multiple centers. Additional evidence is needed to substantiate alterations in functional connectivity between the thalamus and the motor network after a stroke.

The thalamus is a therapeutic target for many diseases, and whether it can become an intervention target for poststroke motor rehabilitation warrants further exploration. Comprising nuclei with diverse cell structures and functions, the thalamus establishes extensive functional connections with the cerebral cortex and other subcortical structures. Disruptions in thalamic structure and function are linked to numerous neurological and psychiatric disorders (Shine et al. 2023; He et al. 2020). Concurrently, specific thalamic nuclei also serve as regulatory treatment targets for neurological diseases—deep brain stimulation of the ventral intermediate nucleus treats movement disorders (Benabid et al. 1991), the anterior and centromedian nuclei are regulatory targets for refractory epilepsy (Piper et al. 2022), and the ventroposterior lateral nucleus alleviates neuropathic pain (Owen et al. 2006). For poststroke motor rehabilitation, noninvasive repetitive transcranial magnetic stimulation or transcranial direct current stimulation of the primary motor cortex is predominantly utilized (Hensel et al. 2023). As these noninvasive neurostimulation therapies were previously thought to only act on the cortical surface, the deep nuclei of the brain were rarely employed as intervention targets. However, recent findings by Shan et al. (2023) suggest that transcranial alternating current stimulation can effectively influence deep brain nuclei, offering a safe and straightforward method. This opens new opportunities for noninvasive neurostimulation technology applications. Additionally, transcranial‐focused ultrasound stimulation is also a highly promising technology that can modulate deep brain tissues with high spatial resolution (Yuksel et al. 2023). This study found that ipsilesional somatosensory‐thalamic connectivity decreased and correlated with motor function, suggesting that the integrative ability of the thalamus for the sensory functional network may be affected after stroke. Further research into using the thalamus as a therapeutic target to improve motor rehabilitation by enhancing the sensory integration capability merits exploration.

This study has some limitations. First, although we used a combination of ROI‐to‐ROI and seed‐to‐voxel approaches to conduct an in‐depth analysis of functional connectivity between the thalamus and cortex, since rs‐fMRI is based on the BOLD signal principle and we used group‐level functional subregion segmentation of the thalamus, the thalamic functional subregion map only indicates approximate locations of the functional subregions and does not reflect structural fiber connections, cytoarchitectonic features of the subregions, or individual differences in stroke patients. Multimodal investigations will be helpful in clarifying the nature of thalamocortical dysconnectivity. Second, this study included only baseline rs‐fMRI scanning, limiting our ability to observe dynamic changes in brain plasticity during rehabilitation. Future longitudinal studies with multiple scanning time points will be valuable for investigating how changes in thalamocortical functional connectivity correlate with motor function recovery trajectories. Furthermore, this was a single‐center study with subjects mainly suffering from mild to moderate strokes and predominantly male, which may limit the generalizability of our findings. We hope that future large‐scale, multicenter longitudinal cohort studies will further validate the results of this study.

## 5 Conclusions

Our study demonstrates that thalamocortical functional connectivity undergoes plastic changes during the subacute phase after stroke, with ipsilesional somatosensory‐thalamic connectivity correlating with upper extremity motor function. These findings support our original research objectives and provide novel insights into the thalamus's role in motor recovery after stroke. Moreover, this study offers preliminary evidence suggesting the thalamus as a potential therapeutic target for poststroke motor rehabilitation. Further investigations are warranted to validate these findings and fully elucidate the mechanisms underlying thalamic involvement in stroke recovery.

## Author Contributions

**Haojie Zhang**: conceptualization, investigation, writing–original draft, methodology, writing–review and editing, formal analysis, project administration, data curation. **Jun Zhao**: funding acquisition, project administration. **Lingzhong Fan**: methodology, writing–review and editing, supervision. **Chaohong Gao**: software, formal analysis, writing–review and editing. **Fang Li**: investigation, writing–review and editing. **Jingya Liu**: investigation, writing–review and editing. **Chen Bai**: investigation, writing–review and editing. **Xingzhu Li**: investigation, software, writing–review and editing. **Bingjie Li**: writing–review and editing, project administration, supervision. **Tong Zhang**: conceptualization, project administration, funding acquisition, writing–review and editing, methodology, supervision.

## Conflicts of Interest

The authors declare no conflicts of interest.

### Peer Review

The peer review history for this article is available at https://publons.com/publon/10.1002/brb3.70321.

## Supporting information



Supporting Information

## Data Availability

The data supporting the findings of this study and the relevant code applied during the data processing are available from the corresponding author on reasonable request.

## References

[brb370321-bib-0002] Aswendt, M. , N. Pallast , F. Wieters , M. Baues , M. Hoehn , and G. R. Fink . 2021. “Lesion Size‐ and Location‐Dependent Recruitment of Contralesional Thalamus and Motor Cortex Facilitates Recovery After Stroke in Mice.” Translational Stroke Research 12, no. 1: 87–97. 32166716 10.1007/s12975-020-00802-3 PMC7803721

[brb370321-bib-0003] Baker, S. N. , B. Zaaimi , K. M. Fisher , S. A. Edgley , and D. S. Soteropoulos . 2015. “Pathways Mediating Functional Recovery.” Progress in Brain Research 218: 389–412. 25890147 10.1016/bs.pbr.2014.12.010

[brb370321-bib-0004] Balbinot, G. , and C. P. Schuch . 2018. “Compensatory Relearning Following Stroke: Cellular and Plasticity Mechanisms in Rodents.” Frontiers in Neuroscience 12: 1023. 30766468 10.3389/fnins.2018.01023 PMC6365459

[brb370321-bib-0005] Behrens, T. E. , H. Johansen‐Berg , M. W. Woolrich , et al. 2003. “Non‐Invasive Mapping of Connections Between Human Thalamus and Cortex Using Diffusion Imaging.” Nature Neuroscience 6, no. 7: 750–757. 12808459 10.1038/nn1075

[brb370321-bib-0006] Benabid, A. L. , P. Pollak , C. Gervason , et al. 1991. “Long‐Term Suppression of Tremor by Chronic Stimulation of the Ventral Intermediate Thalamic Nucleus.” Lancet 337, no. 8738: 403–406. 1671433 10.1016/0140-6736(91)91175-t

[brb370321-bib-0007] Bernhardt, J. , K. S. Hayward , G. Kwakkel , et al. 2017. “Agreed Definitions and a Shared Vision for New Standards in Stroke Recovery Research: The Stroke Recovery and Rehabilitation Roundtable Taskforce.” International Journal of Stroke 12, no. 5: 444–450. 28697708 10.1177/1747493017711816

[brb370321-bib-0008] Calautti, C. , and J. C. Baron . 2003. “Functional Neuroimaging Studies of Motor Recovery After Stroke in Adults: A Review.” Stroke; A Journal of Cerebral Circulation 34, no. 6: 1553–1566. 10.1161/01.STR.0000071761.36075.A6 12738893

[brb370321-bib-0009] Carlson, H. L. , B. T. Craig , A. J. Hilderley , et al. 2020. “Structural and Functional Connectivity of Motor Circuits After Perinatal Stroke: A Machine Learning Study.” NeuroImage: Clinical 28: 102508. 33395997 10.1016/j.nicl.2020.102508 PMC7704459

[brb370321-bib-0010] Carter, A. R. , S. V. Astafiev , C. E. Lang , et al. 2010. “Resting Interhemispheric Functional Magnetic Resonance Imaging Connectivity Predicts Performance After Stroke.” Annals of Neurology 67, no. 3: 365–375. 20373348 10.1002/ana.21905 PMC2927671

[brb370321-bib-0011] Cheng, H. J. , K. K. Ng , and X. Qian , et al. 2021. “Task‐Related Brain Functional Network Reconfigurations Relate to Motor Recovery in Chronic Subcortical Stroke.” Scientific Reports 11, no. 1: 8442. 33875691 10.1038/s41598-021-87789-5 PMC8055891

[brb370321-bib-0012] Choudhury, S. , A. Shobhana , R. Singh , et al. 2019. “The Relationship Between Enhanced Reticulospinal Outflow and Upper Limb Function in Chronic Stroke Patients.” Neurorehabilitation and Neural Repair 33, no. 5: 375–383. 30913964 10.1177/1545968319836233

[brb370321-bib-0013] Cole, M. W. , S. Pathak , and W. Schneider . 2010. “Identifying the Brain's Most Globally Connected Regions.” Neuroimage 49, no. 4: 3132–3148. 19909818 10.1016/j.neuroimage.2009.11.001

[brb370321-bib-0014] Damoiseaux, J. S. , and M. D. Greicius . 2009. “Greater Than the Sum of Its Parts: A Review of Studies Combining Structural Connectivity and Resting‐State Functional Connectivity.” Brain Structure and Function 213, no. 6: 525–533. 19565262 10.1007/s00429-009-0208-6

[brb370321-bib-0015] Fan, L. , H. Li , J. Zhuo , et al. 2016. “The Human Brainnetome Atlas: A New Brain Atlas Based on Connectional Architecture.” Cerebral Cortex 26, no. 8: 3508–3526. 27230218 10.1093/cercor/bhw157 PMC4961028

[brb370321-bib-0016] Feigin, V. L. , and M. O. Owolabi . 2023. “Pragmatic Solutions to Reduce the Global Burden of Stroke: A World Stroke Organization – Lancet Neurology Commission.” Lancet Neurology 22, no. 12: 1160–1206. 37827183 10.1016/S1474-4422(23)00277-6 PMC10715732

[brb370321-bib-0017] Greene, D. J. , S. Marek , E. M. Gordon , et al. 2020. “Integrative and Network‐Specific Connectivity of the Basal Ganglia and Thalamus Defined in Individuals.” Neuron 105, no. 4: 742–758.e6. 31836321 10.1016/j.neuron.2019.11.012 PMC7035165

[brb370321-bib-0018] Grefkes, C. , and G. R Fink . 2020. “Recovery From Stroke: Current Concepts and Future Perspectives.” Neurological Research and Practice 2: 17. 33324923 10.1186/s42466-020-00060-6 PMC7650109

[brb370321-bib-0019] Hale, J. R. , S. D. Mayhew , K. J. Mullinger , et al. 2015. “Comparison of Functional Thalamic Segmentation From Seed‐Based Analysis and ICA.” Neuroimage 114: 448–465. 25896929 10.1016/j.neuroimage.2015.04.027

[brb370321-bib-0020] Hale, J. R. , T. P. White , S. D. Mayhew , et al. 2016. “Altered Thalamocortical and Intra‐Thalamic Functional Connectivity During Light Sleep Compared With Wake.” Neuroimage 125: 657–667. 26499809 10.1016/j.neuroimage.2015.10.041

[brb370321-bib-0021] He, X. , G. Chaitanya , B. Asma , et al. 2020. “Disrupted Basal Ganglia‐Thalamocortical Loops in Focal to Bilateral Tonic‐Clonic Seizures.” Brain 143, no. 1: 175–190. 31860076 10.1093/brain/awz361 PMC7954388

[brb370321-bib-0022] Hensel, L. , F. Lange , C. Tscherpel , et al. 2023. “Recovered Grasping Performance After Stroke Depends on Interhemispheric Frontoparietal Connectivity.” Brain 146, no. 3: 1006–1020. 35485480 10.1093/brain/awac157 PMC9976969

[brb370321-bib-0023] Hwang, K. , J. M. Shine , J. Bruss , D. Tranel , and A. Boes . 2021. “Neuropsychological Evidence of Multi‐Domain Network Hubs in the Human Thalamus.” Elife 10: e69480. 34622776 10.7554/eLife.69480 PMC8526062

[brb370321-bib-0024] Kawaguchi, Y. 2017. “Pyramidal Cell Subtypes and Their Synaptic Connections in Layer 5 of Rat Frontal Cortex.” Cerebral Cortex 27, no. 12: 5755–5771. 29028949 10.1093/cercor/bhx252

[brb370321-bib-0025] Keser, Z. , E. L. Meier , M. D. Stockbridge , B. L. Breining , R. Sebastian , and A. E. Hillis . 2021. “Thalamic Nuclei and Thalamocortical Pathways After Left Hemispheric Stroke and Their Association With Picture Naming.” Brain Connect 11, no. 7: 553–565. 33797954 10.1089/brain.2020.0831 PMC8558071

[brb370321-bib-0026] Kim, B. , and C. Winstein . 2017. “Can Neurological Biomarkers of Brain Impairment Be Used to Predict Poststroke Motor Recovery? A Systematic Review.” Neurorehabilitation and Neural Repair 31, no. 1: 3–24. 27503908 10.1177/1545968316662708

[brb370321-bib-0027] Lacourse, M. G. , E. L. Orr , S. C. Cramer , and M. J. Cohen . 2005. “Brain Activation During Execution and Motor Imagery of Novel and Skilled Sequential Hand Movements.” Neuroimage 27, no. 3: 505–519. 16046149 10.1016/j.neuroimage.2005.04.025

[brb370321-bib-0028] Langhorne, P. , J. Bernhardt , and G. Kwakkel . 2011. “Stroke Rehabilitation.” Lancet 377, no. 9778: 1693–1702. 21571152 10.1016/S0140-6736(11)60325-5

[brb370321-bib-0029] Lee, J. , H. Kim , J. Kim , H. J. Lee , W. H. Chang , and Y. H. Kim . 2021. “Differential Early Predictive Factors for Upper and Lower Extremity Motor Recovery After Ischaemic Stroke.” European Journal of Neurology 28, no. 1: 132–140. 32881176 10.1111/ene.14494

[brb370321-bib-0030] Lim, J. Y. , M. K. Oh , J. Park , and N. J. Paik . 2020. “Does Measurement of Corticospinal Tract Involvement Add Value to Clinical Behavioral Biomarkers in Predicting Motor Recovery After Stroke?” Neural Plasticity 2020: 8883839. 33354207 10.1155/2020/8883839 PMC7735861

[brb370321-bib-0031] Liu, F. , C. Chen , W. Hong , et al. 2022. “Selectively Disrupted Sensorimotor Circuits in Chronic Stroke With Hand Dysfunction.” CNS Neuroscience & Therapeutics 28, no. 5: 677–689. 35005843 10.1111/cns.13799 PMC8981435

[brb370321-bib-0032] Liu, H. , X. Peng , L. Dahmani , et al. 2020. “Patterns of Motor Recovery and Structural Neuroplasticity After Basal Ganglia Infarcts.” Neurology 95, no. 9:e1174.–e87. 32586896 10.1212/WNL.0000000000010149 PMC7538227

[brb370321-bib-0033] Owen, S. L. F. , A. L. Green , J. F. Stein , and T. Z. Aziz . 2006. “Deep Brain Stimulation for the Alleviation of Post‐Stroke Neuropathic Pain.” Pain 120, no. 1–2: 202–206. 16359796 10.1016/j.pain.2005.09.035

[brb370321-bib-0034] Park, C. H. , W. H. Chang , S. H. Ohn , et al. 2011. “Longitudinal Changes of Resting‐State Functional Connectivity During Motor Recovery After Stroke.” Stroke; A Journal of Cerebral Circulation 42, no. 5: 1357–1362. 10.1161/STROKEAHA.110.596155 PMC3589816 21441147

[brb370321-bib-0035] Peters, D. M. , J. Fridriksson , J. D. Richardson , et al. 2021. “Upper and Lower Limb Motor Function Correlates With Ipsilesional Corticospinal Tract and Red Nucleus Structural Integrity in Chronic Stroke: A Cross‐Sectional, ROI‐Based MRI Study.” Behavioural Neurology 2021: 3010555. 34804258 10.1155/2021/3010555 PMC8601844

[brb370321-bib-0036] Piper, R. J. , R. M. Richardson , G. Worrell , et al. 2022. “Towards Network‐Guided Neuromodulation for Epilepsy.” Brain 145, no. 10: 3347–3362. 35771657 10.1093/brain/awac234 PMC9586548

[brb370321-bib-0056] Plantin, J. , M. Verneau , A. K. Godbolt , et al. 2021. “Recovery and Prediction of Bimanual Hand Use After Stroke.” Neurology 97, no. 7: e706–e719. 34400568 10.1212/WNL.0000000000012366 PMC8377875

[brb370321-bib-0037] Saad, Z. S. , S. J. Gotts , K. Murphy , et al. 2012. “Trouble at Rest: How Correlation Patterns and Group Differences Become Distorted After Global Signal Regression.” Brain Connect 2, no. 1: 25–32. 22432927 10.1089/brain.2012.0080 PMC3484684

[brb370321-bib-0038] Sanders, Z. B. , M. K. Fleming , T. Smejka , et al. 2022. “Self‐Modulation of Motor Cortex Activity After Stroke: A Randomized Controlled Trial.” Brain 145, no. 10: 3391–3404. 35960166 10.1093/brain/awac239 PMC9586541

[brb370321-bib-0039] Shan, Y. , H. Wang , Y. Yang , et al. 2023. “Evidence of a Large Current of Transcranial Alternating Current Stimulation Directly to Deep Brain Regions.” Molecular Psychiatry 28, no. 12: 5402–5410. 37468529 10.1038/s41380-023-02150-8 PMC11041720

[brb370321-bib-0040] Shimura, K. , and T. Kasai . 2002. “Effects of Proprioceptive Neuromuscular Facilitation on the Initiation of Voluntary Movement and Motor Evoked Potentials in Upper Limb Muscles.” Human Movement Science 21, no. 1: 101–113. 11983436 10.1016/s0167-9457(01)00057-4

[brb370321-bib-0041] Shine, J. M. , L. D. Lewis , D. D. Garrett , and K. Hwang . 2023. “The Impact of the human Thalamus on Brain‐Wide Information Processing.” Nature Reviews Neuroscience 24, no. 7: 416–430. 37237103 10.1038/s41583-023-00701-0 PMC10970713

[brb370321-bib-0042] Soulard, J. , C. Huber , S. Baillieul , et al. 2020. “Motor Tract Integrity Predicts Walking Recovery: A Diffusion MRI Study in Subacute Stroke.” Neurology 94, no. 6:e583.–e93. 31896618 10.1212/WNL.0000000000008755

[brb370321-bib-0043] Tu, W. , and W. Wei . 2024. “Trends and Projections in Rehabilitation Demand Across 3 Decades in China (1990−2019).” Journal of Aging and Rehabilitation 1, no. 2: 31–35.

[brb370321-bib-0044] Tu, W. J. , Z. Zhao , P. Yin , et al. 2023. “Estimated Burden of Stroke in China in 2020.” JAMA Network Open 6, no. 3:e231455. 36862407 10.1001/jamanetworkopen.2023.1455 PMC9982699

[brb370321-bib-0045] Usrey, W. M. , and S. M. Sherman . 2019. “Corticofugal Circuits: Communication Lines From the Cortex to the Rest of the Brain.” Journal of Comparative Neurology 527, no. 3: 640–650. 29524229 10.1002/cne.24423 PMC6131091

[brb370321-bib-0046] van der Kemp, J. , W. J. Kruithof , T. C. W. Nijboer , C. A. M. van Bennekom , C. van Heugten , and J. M. A. Visser‐Meily . 2019. “Return to Work After Mild‐to‐Moderate Stroke: Work Satisfaction and Predictive Factors.” Neuropsychol Rehabil 29, no. 4: 638–653. 28441897 10.1080/09602011.2017.1313746

[brb370321-bib-0047] van Meer, M. P. , K. van der Marel , W. M. Otte , J. W. Berkelbach van der Sprenkel , and R. M. Dijkhuizen . 2010. “Correspondence Between Altered Functional and Structural Connectivity in the Contralesional Sensorimotor Cortex After Unilateral Stroke in Rats: A Combined Resting‐State Functional MRI and Manganese‐Enhanced MRI Study.” Journal of Cerebral Blood Flow and Metabolism 30, no. 10: 1707–1711. 20664609 10.1038/jcbfm.2010.124 PMC3023403

[brb370321-bib-0048] Ward, L. M. 2013. “The Thalamus: Gateway to the Mind.” Wiley Interdisciplinary Reviews: Cognitive Science 4, no. 6: 609–622. 26304267 10.1002/wcs.1256

[brb370321-bib-0049] Winstein, C. J. , J. Stein , R. Arena , et al. 2016. “Guidelines for Adult Stroke Rehabilitation and Recovery: A Guideline for Healthcare Professionals From the American Heart Association/American Stroke Association.” Stroke 47, no. 6: e98–e169. 27145936 10.1161/STR.0000000000000098

[brb370321-bib-0050] Woodward, N. D. , and S. Heckers . 2016. “Mapping Thalamocortical Functional Connectivity in Chronic and Early Stages of Psychotic Disorders.” Biological Psychiatry 79, no. 12: 1016–1025. 26248537 10.1016/j.biopsych.2015.06.026 PMC4698230

[brb370321-bib-0051] Woodward, N. D. , H. Karbasforoushan , and S. Heckers . 2012. “Thalamocortical Dysconnectivity in Schizophrenia.” American Journal of Psychiatry 169, no. 10: 1092–1099. 23032387 10.1176/appi.ajp.2012.12010056 PMC3810300

[brb370321-bib-0052] Yuksel, M. M. , S. Sun , C. Latchoumane , et al. 2023. “Low‐Intensity Focused Ultrasound Neuromodulation for Stroke Recovery: A Novel Deep Brain Stimulation Approach for Neurorehabilitation?” IEEE Open Journal of Engineering in Medicine and Biology 4: 300–318. 38196977 10.1109/OJEMB.2023.3263690 PMC10776095

[brb370321-bib-0053] Zhang, D. , A. Z. Snyder , M. D. Fox , M. W. Sansbury , J. S. Shimony , and M. E Raichle . 2008. “Intrinsic Functional Relations Between Human Cerebral Cortex and Thalamus.” Journal of Neurophysiology 100, no. 4: 1740–1748. 18701759 10.1152/jn.90463.2008 PMC2576214

[brb370321-bib-0054] Zhang, D. , A. Z. Snyder , J. S. Shimony , M. D. Fox , and M. E. Raichle . 2010. “Noninvasive Functional and Structural Connectivity Mapping of the Human Thalamocortical System.” Cerebral Cortex 20, no. 5: 1187–1194. 19729393 10.1093/cercor/bhp182 PMC2852505

[brb370321-bib-0055] Zhang, H. , J. Zhao , L. Fan , et al. 2024. “Exploring the Structural Plasticity Mechanism of Corticospinal Tract During Stroke Rehabilitation Based Automated Fiber Quantification Tractography.” Neurorehabilitation and Neural Repair 38, no. 6: 425–436. 38676561 10.1177/15459683241249115

