# Supplementary material for: Somatosensory‐Thalamic Functional Dysconnectivity Associated With Poststroke Motor Function Rehabilitation: A Resting‐State fMRI Study
Source: Brain Behav. 2025 Feb 11;15(2):e70321. doi: 10.1002/brb3.70321 (PMC11813981; doi:10.1002/brb3.70321)
Supplement: Supplementary file 1 — Supporting Information [file BRB3-15-e70321-s001.docx]

# Supplemental Material

### Inclusion and exclusion criteria

The inclusion criteria for stroke patients were: (1) ages of 18–85 years; (2) 8–30 days post-first stroke onset; (3) single subcortical infarction in the middle cerebral artery territory with motor deficits; (4) meeting the diagnostic criteria of ischemic stroke set forth by the World Health Organization[1] and confirmed by cranial CT or MRI; and (5) provision of informed consent. The exclusion criteria for stroke patients were: (1) claustrophobia or contraindications for MRI; (2) non-stroke diagnosis affecting limb function; (3) cognitive impairment using the Mini-Mental State Examination scale (cutoff ≤ 24)[2, 3]; (4) obvious depression symptoms based on the Hamilton Depression scale score (cutoff ≥ 20); and (5) incomplete medical records.

### Image data acquisition

All subjects remained awake with their eyes closed during scanning, using the same MRI scanner (Philips Ingenia 3.0T TX) and identical parameters. To ensure scan quality, each participant's head was securely fixed with foam padding and headphones were worn to reduce head movement and scanner noise. The 8-channel head array coil was used for echo plane imaging to obtain high-resolution structure images. The axis of the image was parallel to the anteroposterior commissure line that covers the whole brain. The rs-fMRI scan parameters were as follows: repetition time (TR) = 2000 ms, echo time (TE) = 30 ms, field of view (FOV) = 240 x 240 mm, voxel = 3.75 × 3.75 × 3.4 mm, flip angle = 90˚, matrix = 64 × 62, scan duration 6 minutes 46 seconds. The parameters of the T1_3D weighted fast spin echo sequence were as follows: TR = 7.6 ms, TE = 3.7 ms, flip angle = 8˚, FOV = 256 × 256 mm, voxel = 1 × 1 × 2 mm, and matrix = 256 × 256, scan duration 4 minutes 14 seconds, yielding 200 volumes per subject.

## Supplemental References

1. Aho K, Harmsen P, Hatano S, Marquardsen J, Smirnov VE, Strasser T. Cerebrovascular disease in the community: results of a WHO collaborative study. Bulletin of the World Health Organization. 1980;58(1):113-30.

2. Folstein MF, Folstein SE, McHugh PR. "Mini-mental state". A practical method for grading the cognitive state of patients for the clinician. J Psychiatr Res. 1975;12(3):189-98.

3. Rovner BW, Folstein MF. Mini-mental state exam in clinical practice. Hosp Pract (Off Ed). 1987;22(1a):99, 103, 6, 10.
